# Positive and Negative Affect Schedule in early COVID-19 pandemic

**DOI:** 10.1038/s41597-023-02371-0

**Published:** 2023-07-13

**Authors:** Celia Andreu-Sánchez, Miguel Ángel Martín-Pascual

**Affiliations:** 1 Neuro-Com Research Group, Audiovisual Communication and Advertising Department, Universitat Autònoma de Barcelona, Barcelona, Spain; 2 Research and Development, Institute of Spanish Public Television (IRTVE), Barcelona, Spain

**Keywords:** Population screening, Human behaviour

## Abstract

The COVID-19 pandemic is the first pandemic in the Information Age. It started in Asia and spread rapidly around the world. As a consequence, millions of people were subject to lockdowns, and traditional media and social media reached more people. Our study, carried out during the lockdown, asked people about their feelings and emotions and included a Positive and Negative Affect Schedule (PANAS). Here, we present the data resulting from that study, which could potentially be reused by psychologists interested in learning about the emotional effects of the COVID-19 pandemic as well as to make comparisons before and after the lockdown period in 2020.

## Background & Summary

In March 2020, a pandemic hit the world^1^. The SARS-CoV-2 coronavirus managed to spread all over the world and infect millions of people, provoking the COVID-19 pandemic. Because of this pandemic situation, numerous countries enacted lockdowns for their populations to prevent the coronavirus from spreading and infecting more people. During lockdown, several habits were changed, such as those related to jobs, with an increasing tendency for telecommuting, and those related to media information^2^. During the first part of the pandemic, uncertainty took hold of most people. As a consequence, people’s feelings and emotions were discussed on social media, and several studies in different areas were carried out^3–5^. However, to our knowledge, no one has carried out a Positive and Negative Affect Schedule (PANAS) on the general population, without any geographic restriction, during the first weeks of the first lockdown during the first wave of the COVID-19 pandemic, in which many people were in the first lockdown of their lives. There are several works that have included a PANAS study to approach mental health related to the COVID-19 pandemic^6^, and our data could be useful for comparisons with them. Some studies have been carried out within a specific area, such as in Germany^7^, India^8^, Spain^9^, or Italy^10^. Even though most of our participants were from Spain, there are also respondents from other countries, such as the USA, Italy, Denmark, Venezuela, Turkey, Ukraine, Croatia, Argentina, Mexico, Portugal, and Colombia, among others. Each one of these studies include, apart from the PANAS, other interesting items that participants were asked. Here, we are including gender, age, qualifications, occupation, current lockdown situation, current city, nationality, how they obtain information, and their sports practice. These items can be cross-referenced with the PANAS itself or with variables registered by other studies.

All these studies that included a PANAS scale chose to approach mental health from the perspective of how it was affected by the COVID-19 pandemic. In fact, mental health in the context of the pandemic has become a research area of great interest, with a large number of publications^11–15^ as well as great social and public impact in the news. Consequently, many countries are implementing policy measures aimed at mitigating the effects of the COVID-19 pandemic on the mental health of their citizens^16^. In this context, sharing this type of data becomes important for the continuing development of our societies. Public and private entities can benefit from the use of the data presented here to develop strategies to manage stressful situations such as that represented by the COVID-19 pandemic.

The PANAS scale was introduced in 1988 to meet the need for reliable and valid positive and negative affect scales^17^. It is a self-reported psychometric measure that can be useful in clinical and community contexts. It contains two 10-item mood scales designed to be internally consistent, largely uncorrelated, and stable in longitudinal studies. One of the 10-item mood scales refers to positive items, while the other refers to negative items. The analysed factors emerge consistently across diverse descriptor sets, time frames, response formats, languages and cultures^18^. In a PANAS study, participants must indicate to what extent they have felt a specific way, on a scale from 1 (very slightly or not at all) to 5 (extremely). The positive items from the mood scale are interested, excited, strong, enthusiastic, proud, alert, inspired, determined, attentive and active, whereas the negative ones are distressed, upset, guilty, scared, hostile, irritable, ashamed, nervous, jittery and afraid. These 20 items are mixed in a unique 20-item scale, with an apparently random presentation of positive and negative items to avoid bias. Positive items are counted together, and negative items are counted together. Scores range from 10 to 50 in each category (positive and negative), with lower scores representing lower levels of that positive or negative category.

After the first version of this PANAS scale^17^, several adaptations have been made, including those in different languages. In this study, we used the PANAS scale by Watson, Clark and Tellegen^17^ in English and the PANAS scale by López-Gómez, Hervás and Vázquez^19^ in Spanish.

**Fig. 1:**
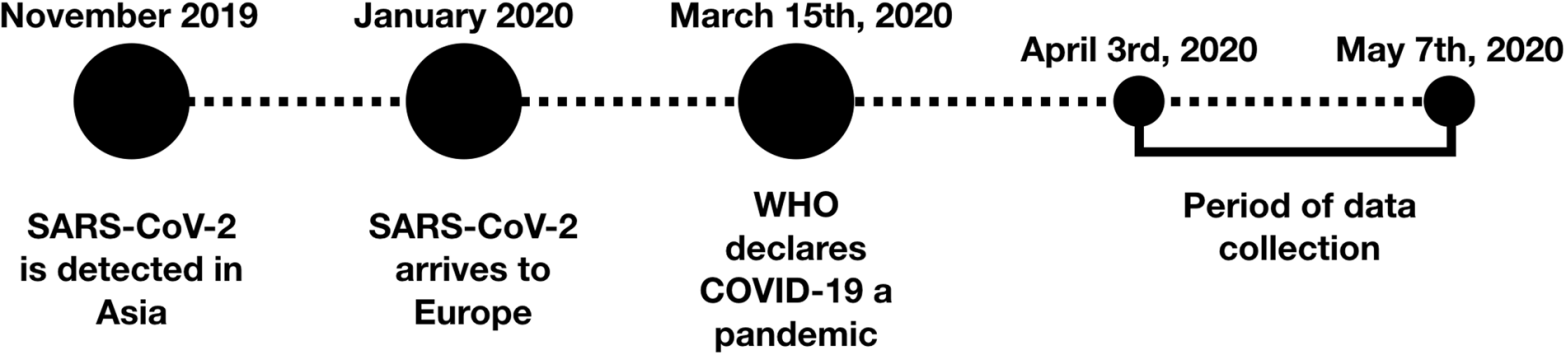
Background of the study design. Timeline since the first report of detection of the SARS-CoV-2 coronavirus and the period of this study’s data collection.

The design of our study (Fig. 1) is related to the global pandemic situation, which started in November 2019 when the SARS-CoV-2 coronavirus was first detected in Asia and before we had planned to do this study. The virus continued to spread globally from January 2020, when it was first detected in Europe. Then, on 15 March 2020, the World Health Organization (WHO) declared COVID-19 to be a pandemic. With that, several Western countries locked down their citizens, as many Eastern countries had already done. This study defined that as its starting point. Between 15 March and 31 March 2020, the study and its distribution strategy were designed, and between 3 April and 7 May 2020, we distributed the study and collected the data.

The goals that motivated us to collect these data were related to this unprecedented and uncertain situation happening collectively in society. As researchers, we were interested in finding out how people were managing their emotions in a world with no real and physical contact due to the lockdown, but in which they were integrated in a digital communication environment.

We believe that one potential use of these data is to analyse the emotional status of people during the first wave of COVID-19. In an uncertain environment where people had not experienced a pandemic situation or a lockdown before, learning about their positive and negative feelings and emotions may be of interest for psychological researchers. In addition, we strongly believe that the potential of these data is in comparing them with other previous and posterior data collected regarding people’s positive and negative feelings and emotions, as well as with other critical situations that may be related or unrelated to COVID-19. For instance, this could be of interest to sociologists and psychologists in the coming decades with the occurrence of other collective disasters or stressful situations. Analysing and comparing these data could also be useful for social and public strategic policies, as well as communication strategies.

## Methods

We designed a study to assess people’s feelings and emotions during the lockdown due to the COVID-19 pandemic. The study was designed to assess participants’ feelings using a Positive and Negative Affect Schedule (PANAS), but it also included information regarding: (1) personal information (gender, age, location, nationality, etc.); (2) how participants remained informed during the lockdown; and (3) sports activities participated in during the lockdown. In addition, participants were presented with images of the SARS-CoV-2 coronavirus and asked about their perception of them, the results of which have been published previously^20^.

### Data acquisition

We distributed the study online in an open approach (not password protected) and sent it to different websites for its promotion. The questionnaire was distributed between 3 April and 7 May 2020. The survey took approximately 15–20 minutes to complete. Data were obtained from 333 participants; 41 of them answered the questionnaire in English, and the rest answered in Spanish. The questionnaire protocol was as follows: (1) subject’s informed consent was requested; (2) participants introduced personal data, maintaining anonymity; (3) participants were asked about their media habits during the pandemic, the sport activities that they practised, and then completed the PANAS scale regarding their emotional positive and negative feelings; and (4) participants were asked to define their perception of 46 images representing the SARS-CoV-2 coronavirus, the results of which demonstrate how the images’ attributes affect people’s perception of the virus; as mentioned above, these have already been published^20^ and thus are not included here.

**Fig. 2:**
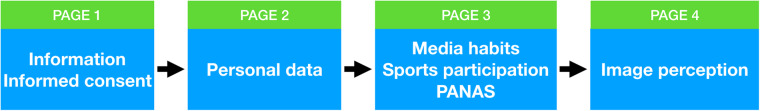
Overview of study protocol. The present paper includes data from pages 1, 2 and 3, with data from page 4 available elsewhere^20^.

The study was carried out using Google Forms, with a four-page protocol (Fig. 2). The first page included information regarding the research objectives of the study and the informed consent. By clicking the NEXT button, participants gave their consent to participate in the study. The second page asked for personal data. Participants were asked for the following items: gender, age range, educational level, job, their lockdown situation, current city, and nationality. The third page included information related to their media habits, sports activities and the PANAS. Finally, the fourth page included questions regarding the perception of 46 images representing SARS-CoV-2. At the end of the last page, participants clicked the SEND button and the data were sent to the researchers’ online database.

### Data pre-processing

After closing the questionnaire, raw data from all participants were analysed and tabulated in a spreadsheet. Colour codification was used to distinguish the different sections of the study: personal data, media habits, sports participation, the PANAS and image perception. Within the PANAS section, we used two colours to distinguish the positive from the negative questions. In the Likert questions, the study was designed with a 1–5 scale (1, very slightly or not at all; 2, a little; 3, moderately; 4, quite a bit; 5, extremely). Data in the records follow this codification.

333 participants answered the questionnaire. 25 of them did it in English, the rest in Spanish. 316 were from Europe, 1 were from North America and 16 were from South America. 1 participant was from Russia, 1 from Iran and 2 from Turkey. Note that some people indicated double nationality. There were no participants from other regions. 142 responders were males (42.64%), 189 were women (56.76%) and 2 preferred not to say (0.6%). Preliminary results show that: (1) participants were more informed by digital newspapers and Government press conferences; (2) most people (70.57%) spent more time sitting/lying down than before, also many of them (68.17%) thought they were eating more than they used to, while most of them (70.27%) felt more motivated to do sports, and almost every one (94.59%) indicated that they watched more sports on television; and (3) the positive emotions of the PANAS obtained a higher mean score (2.91) than the negative ones (2.07). Further analysis should be done the data to achieve proper significant results.

### Ethics approval

This study obtained ethical approval from the Ethics Committee on Animal and Human Experimentation (CEEAH) of the Universitat Autònoma de Barcelona (Spain, reference no. CEEAH 5109). Participants confirmed that they were 18 years of age or older when offering their consent in the online questionnaire. This study was carried out online due to the lockdown situation, which prevented us from distributing the questionnaire personally. This online formula to obtain personal consent was approved by the University Ethics Committee. Participation was voluntary, and data were obtained in accordance with the tenets of the Declaration of Helsinki and other relevant international codes and guidelines.

## Data Records

The latest version of the data was updated on 22 May 2020. The dataset is available online^21^ (10.5281/zenodo.8057093). We describe the dataset fields in detail below. We share the dataset in English and Spanish; each version is in a clearly marked sheet.

### Questionnaire

The questions were distributed in different sections, as mentioned above: personal data, media habits, sports participation and the PANAS. The final section that includes the perception of 46 images representing SARS-CoV-2 is not shared here as it is available elsewhere^20^.

The personal data section included information regarding gender, age, education level, occupation, current lockdown situation, current city, and nationality (Table 1). The data regarding media habits include a question regarding the media that participants use to stay informed (Table 2). The data about sports participation consist of a question regarding the frequency with which participants practised any kind of sport (Table 3), and the PANAS scale was presented next (Table 4).

**Table 1:** Personal data.

Gender	• Man • Woman • Other • I prefer not to say
Age, years	• 18–25 • 26–35 • 36–45 • 46–55 • 56–65 • +65
Education level	• Completed primary education • Completed secondary education • Completed trade/technical training • Completed college/university
Occupation	
During the COVID-19 pandemic, I am currently in a situation of…(*Select all that apply*.)	• Lock down • Preventive quarantine • Working from home • Attending my workplace regularly • Covid-19 affected • Other
What city are you in now?	
What is your nationality?

**Table 2:** Media habits.

During the COVID-19 pandemic or the period of social isolation due to COVID-19, you stay informed through: *1* = *Very slightly or not at all*/2 = *A Little*/3 = *Moderately*/4 = *Quite a bit*/5 = *Extremely*					
	1	2	3	4	5
Television					
Family and friends					
Radio					
Messages whose authorship I do not know that come to me through WhatsApp					
Newspapers					
Co-workers					
Digital newspapers					
Comments on social media from people I don’t know					
Neighbours					
Government press conferences

**Table 3:** Sports participation.

Since the COVID-19 pandemic or period of social isolation due to COVID-19 was declared:		
	Yes	No
I do sports regularly		
I spend more time sitting/lying down than before		
I have adapted my sports routines		
I think I’m eating more than I used to		
I am more motivated to do sports		
I watch more sports on television

**Table 4:** The PANAS scale.

Indicate to what extent you feel these emotions since the COVID-19 pandemic or period of social isolation due to COVID-19 was declared: 1 = *Very slightly or not at all*/2 = *A little*/3 = *Moderately*/4 = *Quite a bit*/5 = *Extremely*						
		1	2	3	4	5
1	Interested					
2	Distressed					
3	Excited					
4	Upset					
5	Strong					
6	Guilty					
7	Scared					
8	Hostile					
9	Enthusiastic					
10	Proud					
11	Irritable					
12	Alert					
13	Ashamed					
14	Inspired					
15	Nervous					
16	Determined					
17	Attentive					
18	Jittery					
19	Active					
20	Afraid					

### Main dataset

The dataset consists of a spreadsheet with two pages. Both have the same structure and information, but in different languages, with page 1 in English and page 2 in Spanish. The data are distributed with different items in each column and different participants in each row. Most of the presented variables in each column are self-explanatory; however, note that in the case where numbers between 1 and 5 are given as answers, these correspond to questions asked with a Likert scale value: 1, very slightly or not at all; 2, a little, 3, moderately; 4, quite a bit; 5, extremely.

## Technical Validation

Here we used two PANAS scales, one in Spanish and another in English. Both scales were validated in their own languages by previous authors.

The scale^17^ was originally published in English and initially validated by Watson, Clark and Tellegen in 1988. The authors developed several ways to approach the technical validation of the test, including a normative and reliability approach to the data (basic scale data, test–retest reliability, generalizability to non-student samples), factorial validity (scale validity, item validity, rating scale effects) and external validity (correlations with measures of distress and psychopathology, intraindividual analyses of non-test correlates).

The Spanish scale^19^ was developed in 2015 on the basis of the English version^17^. The first step taken consisted in translating the original terms into Spanish. To do so, authors López-Gómez, Hervás and Vázquez followed recommendations for the translation and cultural adaptation of self-reported outcomes measures^22^. They developed the translation with the help of eight psychologists, two from Spain and six from different Spanish-speaking countries in Latin America, with the aim of utilizing terms that could be properly and equally understood by all Spanish-speaking users. To validate their data, the authors also applied different approaches, including exploratory factorial analysis, confirmatory factorial analysis, factorial invariance analysis and the relationships among variables. They obtained an even higher Cronbach coefficient for the positive and negative items than the original scale.

The acquisition of these data were developed in the initial weeks of the first wave of COVID-19 in Europe and America. Due to the limitations that lockdowns imposed on people, the distribution of the data was limited. The data was collected with convenience using snowball sampling. We did not do any checking for poor responding. The Cronbach alpha for the Spanish^19^ version of the PANAS, was 0.92 for the PA and 0.88, for the NA. The alpha reliabilities of the English^17^ version of the PANAS was of 0.86 for the PA and 0.87 for the NA scales. The technical validation of the scale in both languages (Spanish and English), as well as the exceptional circumstances of that time, make these datasets valuable for present and future studies.

## Usage Notes

There are very little data on the impact that the SARS-Cov-2 pandemic had on people’s emotions and routines during the early stages of lockdown. In 2023, when the peak of COVID-19 infection incidence seems to have passed, and when daily routines from 2020 have more or less resumed, it is interesting to compare these data with people’s current experiences and practices. In the so-called return to normality, the concept of “mental health” seems to have acquired a new meaning in the post-pandemic world. Additionally, the incidence of mental disorders in young generations is increasing, and this increase could be related to the period of lockdown and the forced change in routine. A persistent post-COVID-19 psychological crisis could even be an avenue for future research^23^.

All the literature reviews on public mental health coincide in their indication of anxiety and depression as the most prominent symptoms among those affected by the coronavirus, but they are generally studies with infected patients and those in hospitals^24^. There are far fewer studies on sleep problems, substance abuse and domestic violence during lockdown^25^, which also seem to have increased incidence in childhood and youth in particular^26^. Comparative studies of pre- and post-COVID-19 routines are needed to improve our understanding of this worsening trend in public mental health.

Finally, a weakness of the data presented can be seen in the results of the PANAS and a small bias in its population, as it presents a majority of responses from people with high levels of education and from university settings. This is due to how the survey was distributed at the beginning of lockdown. Reliable broadcast channels were sought, and these were often linked to academia or university or scientific institutions. However, we believe that the perceptions and routines in lockdown obtained in this study have interesting value, especially regarding prospective and possible comparative studies.

## Data Availability

No custom code was used in this study. We used Google Forms to create the questionnaire and manage the data. We used Microsoft Excel to tabulate and distribute the data.
